# Ascariasis in Humans and Pigs on Small-Scale Farms, Maine, USA, 2010–2013

**DOI:** 10.3201/eid2102.140048

**Published:** 2015-02

**Authors:** Leigh Ann Miller, Kate Colby, Susan E. Manning, Donald Hoenig, Elizabeth McEvoy, Susan Montgomery, Blaine Mathison, Marcos de Almeida, Henry Bishop, Alexandre Dasilva, Stephen Sears

**Affiliations:** Maine Center for Disease Control and Prevention, Maine Department of Health and Human Services, Augusta, Maine, USA (L.A. Miller, K. Colby, S.E. Manning, S. Sears);; Centers for Disease Control and Prevention, Atlanta, Georgia, USA (L.A. Miller, S.E. Manning, S. Montgomery, B. Mathison, M. de Almeida, H. Bishop, A. Dasilva);; University of Southern Maine, Portland, Maine, USA (K. Colby);; Maine Department of Agriculture, Conservation and Forestry, Augusta (D. Hoenig, E. McEvoy)

**Keywords:** Ascariasis, Ascaris lumbricoides, Ascaris suum, humans, pigs, Maine, USA, parasites, nematodes, helminths

## Abstract

*Ascaris* is a genus of parasitic nematodes that can cause infections in humans and pigs. During 2010–2013, we identified 14 cases of ascariasis in persons who had contact with pigs in Maine, USA. *Ascaris* spp. are important zoonotic pathogens, and prevention measures are needed, including health education, farming practice improvements, and personal and food hygiene.

*Ascaris* spp. are parasitic nematodes whose eggs can remain infective in the environment for years. Ascariasis, infection with *Ascaris* spp., results from ingestion of infective eggs (*1*). *A. lumbricoides* nematodes are among the most prevalent human parasites worldwide, infecting >1 billion persons globally (*2*). Most human *Ascaris* infections are asymptomatic, but symptoms can include acute lung inflammation, abdominal distension and pain, and intestinal obstruction (*2*). Pigs are infected with *A. suum* nematodes, and symptoms can include coughing or thumping, liver damage, impaired growth, and increased susceptibility to other infections (*3*).

The overall extent of ascariasis in human and pigs in the United States, and in Maine, is unknown because the infection is not nationally notifiable or reportable at the state level. Ascariasis and other soil-transmitted helminth infections were highly prevalent in the southern United States and Appalachia as recently as the 1980s and were largely attributable to poor sanitation and poverty (*4**,**5*). Less is known about ascariasis prevalence elsewhere in the United States, including the Northeast, but infection has been presumed to be uncommon.

Experimental cross-transmission studies have demonstrated that *A. lumbricoides* can infect pigs and that *A. suum* can infect humans (*3**,**6*). Studies have also indicated that pigs are the main source of human *Ascaris* infections in areas considered to have no or low *Ascaris* prevalence (*7*–*9*). We describe 14 human cases of ascariasis associated with contact with pigs at 7 farms in Maine, USA, during 2010–2013 (*10*). In particular, we highlight an investigation at 1 farm (farm X).

## The Study

The 14 human ascariasis cases were reported by human and animal health care providers to the Maine Department of Health and Human Services (DHHS) or the Maine Department of Agriculture, Conservation and Forestry. Maine DHHS staff interviewed patients from each farm. Patients with confirmed infection had excreted in stool ≥1 worm that was subsequently laboratory-identified as *Ascaris* sp. Patients with probable infection reported excreting ≥1 worm in stool and were epidemiologically associated with a confirmed patient. Patients with suspected infection were persons with symptoms consistent with larval migration who had been on the same implicated farm as a patient with confirmed infection or persons who excreted ≥1 worm in stool without laboratory confirmation or association with a patient with confirmed illness.

The Figure displays geographic information regarding the 14 human cases (8 confirmed, 4 probable, and 2 suspected) from 7 unrelated small-scale farms in 6 counties in Maine. Three of the 7 farms raised organic vegetables. Workers or residents did not rotate among farms, but all 14 case-patients reported contact with pigs. Ten (71%) patients reported no international travel history. Of the 4 patients with a history of international travel, 2 reported receiving previous treatment for parasites. The Table shows the location of farms where infections were found, the laboratory results, and case classifications made during April 2010–March 2013. Patients were 1–53 years of age (median 25 years); 93% were female. The 3 pediatric patients were children who resided at 2 of the farms.

**Figure:**
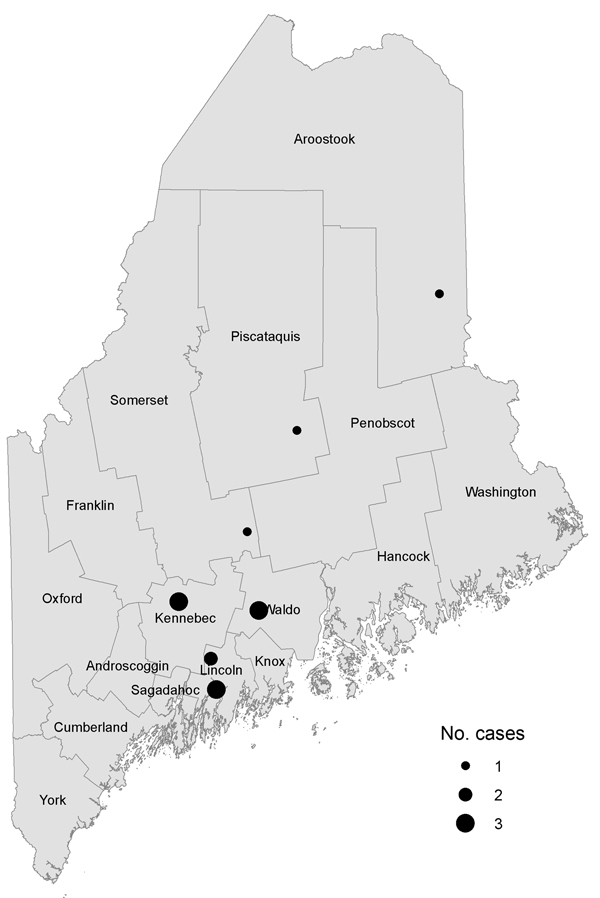
Locations of human ascariasis cases in Maine, USA, 2010–2013.

**Table:** Sample source information, results of laboratory testing, and case classification for *Ascaris* spp. nematode infections in humans and pigs on 7 farms, Maine, USA, 2010–2013*

Farm location and source	Sample type and year collected	Testing laboratory	Testing result	Case classification
Aroostook County				
Woman, age 25 y	NA			Suspected
Kennebec County				
Woman, age 21 y	Worms shed in feces, 2010	Private laboratory	*Ascaris* sp.	Confirmed
Woman, age 26 y	Worms shed in feces, 2010	Private laboratory	*Ascaris* sp.	Confirmed
Woman, age NA	NA			Suspected
Lincoln County†				
Boy, age 3 y	Worms shed in feces, 2012	CDC	*Ascaris* sp.	Confirmed
Woman, age 31 y	Worms shed in feces, 2012	CDC	*Ascaris* sp.	Confirmed
** Woman, age 29 y**	**Worms shed in feces, 2012**	**CDC**	***Ascaris* sp.**	**Confirmed**
** Woman, age 36 y**	**NA**			**Probable**
** Woman, age 25 y**	**NA**			**Probable**
** Farm X pigs**	**Pooled pig feces, 2012**	**Cornell**	***A. suum* eggs**	**NA**
** Farm X pigs at slaughter**	**5 worms collected from pigs, 2012**	**CDC**	***Ascaris* sp.**	**NA**
Piscataquis County				
Woman, age 53 y	Worms shed in feces, 2013	CDC	*Ascaris* sp.	Confirmed
Pig from farm	Pooled pig feces, 2013	Cornell	*A. suum* eggs	NA
Somerset County				
Woman, age 24 y	Worms shed in feces, 2012	Private laboratory	*Ascaris* sp.	Confirmed
Waldo County				
Woman, age 29 y	Worms shed in feces, 2012	CDC	*Ascaris* sp.	Confirmed
Girl, age 4 y	NA			Probable
Girl, age 2 y	NA			Probable

*Boldface indicates samples from farm X. CDC, Centers for Disease Control and Prevention; Cornell, Cornell University Animal Health Diagnostic Center; NA, not available. †Two farms were located in Lincoln County.

In October 2012, Maine DHHS conducted a site visit of farm X. A pooled fecal specimen was collected from 10 pigs. A worm specimen from a person was sent to the Centers for Disease Control and Prevention (CDC) for identification. Pooled pig feces and pig and human worm specimens were sent to private, university, and CDC laboratories for identification.

Farm X grew and sold organic vegetables and raised conventional and organic livestock, including dairy cows, laying hens, sheep, and pigs. Among 12 persons who worked at farm X during the fall of 2012, a total of 3 ascariasis cases occurred; all patients had gastrointestinal illness onsets after May 2012 and were treated with albendazole. Of these 3 patients, 2 reported travel in Africa during the previous year. One of these patients, whose *Ascaris* spp. infection was confirmed, had 3 stool samples reportedly test negative for parasites in February 2012 before returning to United States. The other patient who reported international travel and who had probable *Ascaris* spp. infection was reportedly treated for unspecified parasites in December 2011 while abroad. The third patient, who also had probable *Ascaris* spp. infection, reported travel in Asia within the previous 2 years but was not previously screened or treated for parasitic infections.

In October 2012, worms were recovered from 5 of 10 pigs from farm X at slaughter. Farm X had purchased these pigs as piglets from a local supplier in May 2012. During July 2012, the animals were treated for cough with dichlorvos. Farm X periodically rotates pig pens and vegetable gardens to different locations. These pigs were penned where pigs had been raised 1.5 years previously, in an area ≈15 feet from active vegetable plots. A mixture of hay used as bedding material for pigs, and pig manure was used as fertilizer for growing vegetables. Three hand-washing stations were observed at farm X.

At farm X, we detected *Ascaris* eggs in pooled feces by zinc sulfate and sugar flotation testing methods. Human and pig worm specimens collected at farm X were confirmed as *Ascaris* spp. at CDC. We were unable to determine if pigs from any of the 7 affected farms had a source in common (e.g., common swine stock or breeder).

## Conclusions

We determined that direct or indirect exposure to pigs was the single common factor in all 14 cases of human ascariasis we investigated. At farm X, where detailed information was available regarding pig husbandry, the timing of illness among the farm workers was consistent with acquisition of *Ascaris* infection from pigs. Cross-transmission of *Ascaris* infections between humans and pigs likely occurred on these farms. 

Ascariasis occurred at multiple locations in Maine where farm workers had no history of travel to parts of the world where transmission occurs, strengthening our position that pigs introduced infection. We believe that *Ascaris* eggs persisted in farm soil for extended periods, which led to ongoing transmission (*11*). Laboratory rRNA analyses of the pig and human isolates contribute to a growing body of evidence that *A. lumbricoides* and *A. suum* are genetically very closely related and might, in fact, be a single species (*12*–*14*)*.*

Certain farm practices might have contributed to human exposure to *Ascaris* eggs, including, as noted at farm X, use of pig manure as fertilizer, use of pig bedding for compost, and location of pig pens near where produce is grown. None of the 7 farms we investigated managed pigs according to organic farming standards, and pigs were not regularly dewormed. The human infections occurred on Maine farms with limited numbers (<30) of pigs, where farmers might be unaware of their risk for acquiring ascariasis. Maine does not have a wild pig population; therefore, wild pigs were not a potential source of infection or environmental contamination.

Recommendations to reduce transmission of *Ascaris* spp. nematodes include keeping pig pens separate from vegetable fields and avoiding use of pig manure for fertilizer, especially on produce. Ideally, farms should have dedicated equipment for handling animal waste and stall cleaning. Farm workers should wash hands before and after contact with pigs, pig waste, or soil contaminated with pig waste. Because *Ascaris* eggs can remain viable for extended periods in soil, raw produce should be washed thoroughly before consumption. For optimal animal health, pigs should be dewormed before introduction to the farm and should be regularly dewormed, and humans and pigs should be treated concurrently when human cases occur. Preventing *Ascaris* infections requires an integrated, OneHealth approach that addresses on-farm practices, animal husbandry, and health education efforts.
